# Correction: Hormetic and transgenerational effects in spotted-wing Drosophila (Diptera: Drosophilidae) in response to three commonly-used insecticides

**DOI:** 10.1371/journal.pone.0317051

**Published:** 2024-12-31

**Authors:** Carrie Deans, William D. Hutchison

Fig 2 is incorrect. Figs 2 and 3 are the same. Please see the corrected version of Fig 2 here.

**Fig 2:**
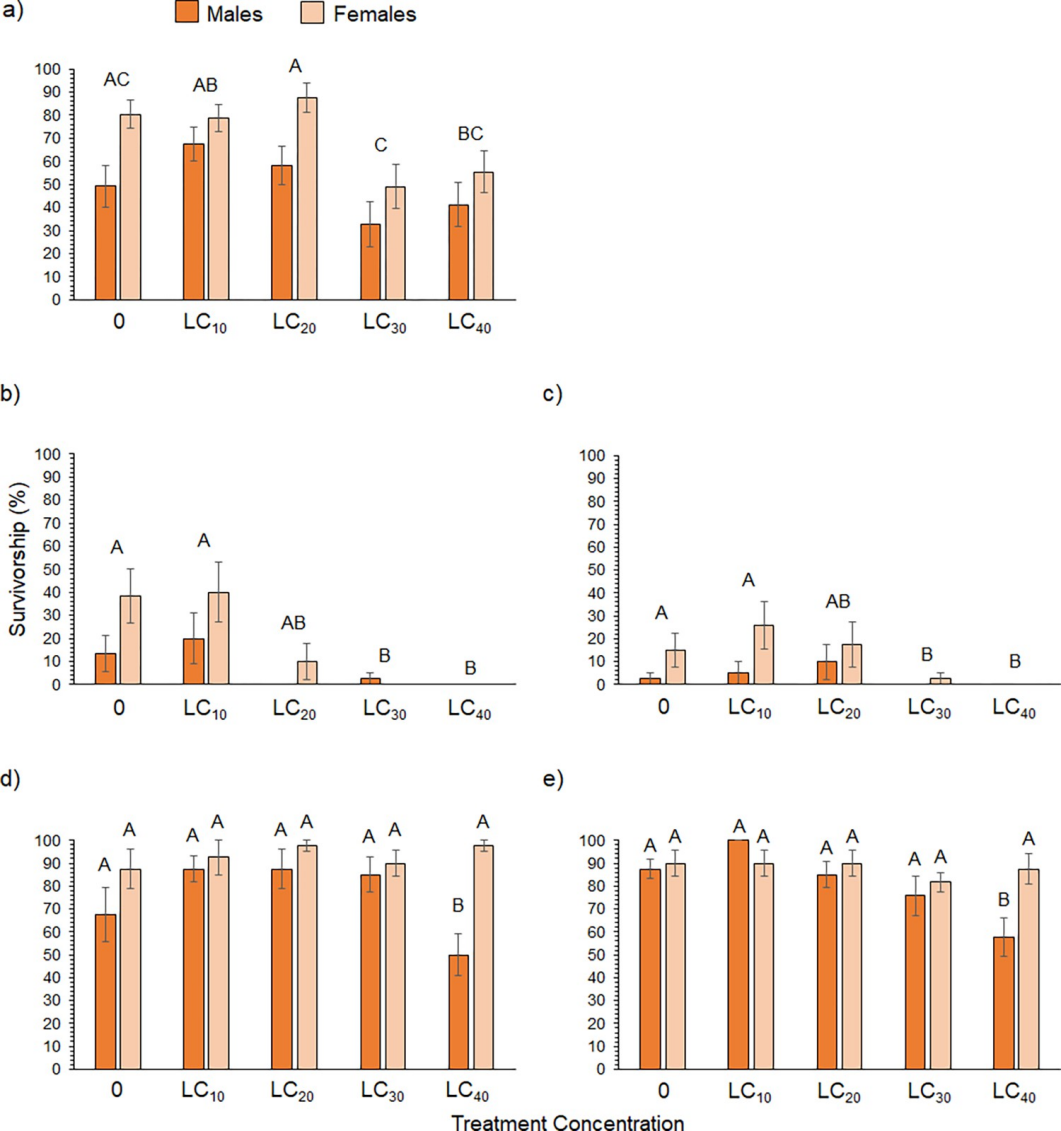
Survivorship. Average survivorship for male and female flies across sub-lethal concentrations of (a) zeta-cypermethrin, (b) single-exposure and (c) double-exposure to spinetoram, (d) single-exposure and (d) double-exposure to pyrethrin (N = 10). Different letters indicate significant post-hoc differences between treatments or sex where significant main effects or interactions were found (S1 and S2 Tables; Tukey’s Test, P ≤ 0.05).

Consequently, in Table 1, the value in R^2^ (last column) of Insecticide zeta- cypermethrin (first row) is incorrect. It should have been 0.842. Please see the correct Table 1 here.

**Table 1:** Statistics for the initial dose-response probit analysis for each insecticide.

Insecticide	X^2^	df	P-value	slope	intercept	R^2^
zeta-cypermethrin	0.141	2	0.932	0.444	4.702	0.842
spinetoram	0.231	3	0.972	0.549	4.370	0.812
pyrethrin	0.700	2	0.705	0.725	5.817	0.972

Moreover, in the Hormetic effects subsection of Results, there is an error in the second sentence of the first paragraph. The correct sentence is: Overall, female survivorship was 1.4 times higher than that of male flies.

## References

[1] Deans C, Hutchison WD (2022) Hormetic and transgenerational effects in spotted-wing Drosophila (Diptera: Drosophilidae) in response to three commonly-used insecticides. PLOS ONE 17(7): e0271417. 10.1371/journal.pone.0271417 35862486 PMC9302851

