# Supplementary figures and images for: Hormetic and transgenerational effects in spotted-wing Drosophila (Diptera: Drosophilidae) in response to three commonly-used insecticides
Source: PLoS One. 2022 Jul 21;17(7):e0271417. doi: 10.1371/journal.pone.0271417 (PMC9302851; doi:10.1371/journal.pone.0271417)

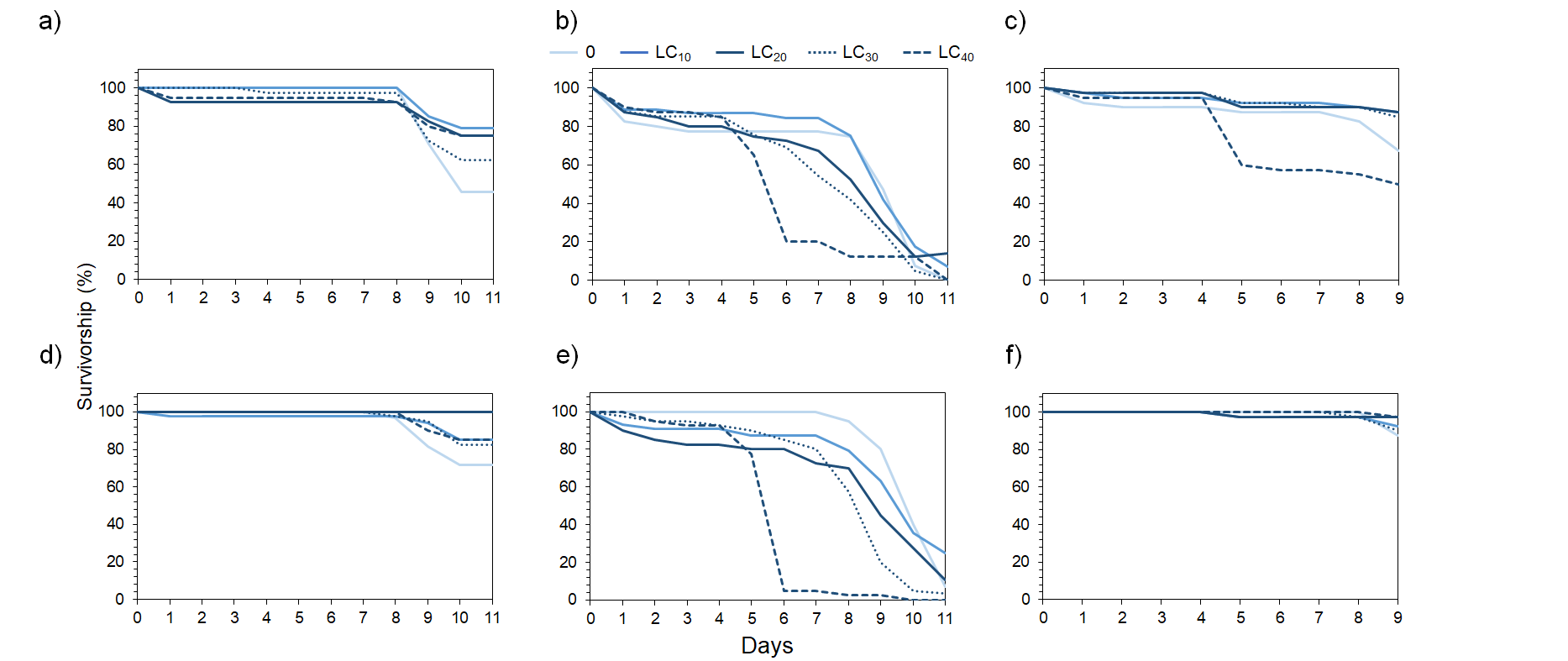

Supplement: S1 Fig — Survival curves for male (a-c) and female (d-f) flies from the (a, d) zeta-cypermethrin, (b, e) spinetoram, and (c, f) pyrethrin experiments (N = 10). (TIF) [file pone.0271417.s001.tif]
