# Supplementary material for: Hormetic and transgenerational effects in spotted-wing Drosophila (Diptera: Drosophilidae) in response to three commonly-used insecticides
Source: PLoS One. 2022 Jul 21;17(7):e0271417. doi: 10.1371/journal.pone.0271417 (PMC9302851; doi:10.1371/journal.pone.0271417)
Supplement: S1 Table — Tukey’s post-hoc test P-values for the significant treatment effects on survival from the three-way ANOVA. Bolded values indicate statistically significant P-values (P-value ≤ 0.05). (PDF) [file pone.0271417.s002.pdf]

SI Table 1. Survivorship post-hoc results for treatment effects. Tukey's post-hoc test P-values for the significant treatment effects on survival from the three-way ANOVA. Bolded values indicate statistically significant P-values (P-value ≤ 0.05).

Contrasts	zeta-cypermethrin	spinetoram	pyrethrin
treatment LC_0 vs	0.903	0.984	0.254
LC_0 vs LC_{20}	0.805	0.207	0.673
LC_0 vs LC_{30}	0.070	**0.002**	0.964
LC_0 vs LC_{40}	0.420	**<0.0001**	0.293
LC_{10} vs LC_{20}	0.999	0.062	0.955
LC_{10} vs LC_{30}	**0.005**	**<0.0001**	0.058
LC_{10} vs LC_{40}	0.073	**<0.0001**	**0.001**
LC_{20} vs LC_{30}	**0.002**	0.466	0.273
LC_{20} vs LC_{40}	**0.041**	0.185	**0.011**
