# Supplementary material for: Hormetic and transgenerational effects in spotted-wing Drosophila (Diptera: Drosophilidae) in response to three commonly-used insecticides
Source: PLoS One. 2022 Jul 21;17(7):e0271417. doi: 10.1371/journal.pone.0271417 (PMC9302851; doi:10.1371/journal.pone.0271417)
Supplement: S2 Table — Tukey’s post-hoc test P-values for the significant treatment effects on eclosion success for the spinetoram treatment. Bolded values indicate statistically significant P-values (P-value ≤ 0.05). (PDF) [file pone.0271417.s003.pdf]

**SI Table 2. Eclosion success post-hoc results for spinetoram**. Tukey's post-hoc test P-values for the significant treatment effects on eclosion success for the spinetoram treatment. Bolded values indicate statistically significant P-values (P-value ≤ 0.05).

| Contrasts | spinetoram |
|---|---|
| treatment $LC_0$ vs | 0.496 |
| $LC_0$ vs $LC_{20}$ | 0.842 |
| $LC_0$ vs $LC_{30}$ | 0.976 |
| $LC_0$ vs $LC_{40}$ | 0.394 |
| $LC_{10}$ vs $LC_{20}$ | 0.976 |
| $LC_{10}$ vs $LC_{30}$ | 0.199 |
| $LC_{10}$ vs $LC_{40}$ | **0.012** |
| $LC_{20}$ vs $LC_{30}$ | 0.496 |
| $LC_{20}$ vs $LC_{40}$ | 0.053 |
| $LC_{30}$ vs $LC_{40}$ | 0.759 |
