# Supplementary material for: Hormetic and transgenerational effects in spotted-wing Drosophila (Diptera: Drosophilidae) in response to three commonly-used insecticides
Source: PLoS One. 2022 Jul 21;17(7):e0271417. doi: 10.1371/journal.pone.0271417 (PMC9302851; doi:10.1371/journal.pone.0271417)
Supplement: S3 Table — Tukey’s post-hoc test P-values for the significant treatment*exposure interaction on adult mass for the spinetoram treatment. Bolded values indicate statistically significant P-values (P-value ≤ 0.05). (PDF) [file pone.0271417.s004.pdf]

SI Table 3. Treatment*exposure post-hoc results for spinetoram. Tukey's post-hoc test P-values for the significant treatment*exposure interaction on adult mass for the spinetoram treatment. Bolded values indicate statistically significant P-values (P-value ≤ 0.05).

treatment by exposure		
contrasts	acute	chronic
LC_0 vs LC_{10}	1.00	1.00
LC_0 vs LC_{20}	1.00	1.00
LC_0 vs LC_{30}	1.00	**0.024**
LC_0 vs LC_{40}	1.00	0.736
LC_{10} vs LC_{20}	1.00	1.00
LC_{10} vs LC_{30}	0.658	**0.019**
LC_{10} vs LC_{40}	1.00	0.656
LC_{20} vs LC_{30}	1.00	**0.001**
LC_{20} vs LC_{40}	1.00	0.113
LC_{30} vs LC_{40}	1.00	1.00
exposure by treatment		
treatment	acute vs chronic	
LC_0	0.167	
LC_{10}	0.061	
LC_{20}	**0.011**	
LC_{30}	**0.002**	
LC_{40}	0.634	
