# Supplementary material for: Hormetic and transgenerational effects in spotted-wing Drosophila (Diptera: Drosophilidae) in response to three commonly-used insecticides
Source: PLoS One. 2022 Jul 21;17(7):e0271417. doi: 10.1371/journal.pone.0271417 (PMC9302851; doi:10.1371/journal.pone.0271417)
Supplement: S4 Table — Tukey’s post-hoc test results for the significant sex*treatment interaction on lifespan for the pyrethrin treatment. Bolded values indicate statistically significant P-values (P-value ≤ 0.05). (PDF) [file pone.0271417.s005.pdf]

SI Table 4. Survivorship post-hoc results for pyrethrin. Tukey's post-hoc test results for the significant sex*treatment interaction on lifespan for the pyrethrin treatment. Bolded values indicate statistically significant P-values (P-value ≤ 0.05).

	treatment by sex	
contrasts	males	females
LC_0 vs LC_{10}	0.144	1.00
LC_0 vs LC_{20}	1.00	1.00
LC_0 vs LC_{30}	1.00	1.00
LC_0 vs LC_{40}	**0.015**	1.00
LC_{10} vs LC_{20}	1.00	1.00
LC_{10} vs LC_{30}	0.347	0.930
LC_{10} vs LC_{40}	**<0.0001**	1.00
LC_{20} vs LC_{30}	1.00	0.657
LC_{20} vs LC_{40}	**<0.0001**	1.00
LC_{30} vs LC_{40}	**0.005**	0.833
	sex by treatment	
treatment	male vs female	
LC_0	0.059	
LC_{10}	0.870	
LC_{20}	0.248	
LC_{30}	0.783	
LC_{40}	**<0.0001**	
