# Supplementary material for: Hormetic and transgenerational effects in spotted-wing Drosophila (Diptera: Drosophilidae) in response to three commonly-used insecticides
Source: PLoS One. 2022 Jul 21;17(7):e0271417. doi: 10.1371/journal.pone.0271417 (PMC9302851; doi:10.1371/journal.pone.0271417)
Supplement: S5 Table — Tukey’s post-hoc test P-values for the significant treatment effects on pupal rate for the pyrethrin treatment. Bolded values indicate statistically significant P-values (P-value ≤ 0.05). (PDF) [file pone.0271417.s006.pdf]

**SI Table 5. Pupal rate post-hoc results for pyrethrin**. Tukey's post-hoc test P-values for the significant treatment effects on pupal rate for the pyrethrin treatment. Bolded values indicate statistically significant P-values (P-value ≤ 0.05).

| Contrasts | pyrethrin |
|---|---|
| treatment $LC_0$ vs | 0.254 |
| $LC_0$ vs $LC_{20}$ | 0.673 |
| $LC_0$ vs $LC_{30}$ | 0.964 |
| $LC_0$ vs $LC_{40}$ | 0.293 |
| $LC_{10}$ vs $LC_{20}$ | 0.955 |
| $LC_{10}$ vs $LC_{30}$ | 0.058 |
| $LC_{10}$ vs $LC_{40}$ | **0.001** |
| $LC_{20}$ vs $LC_{30}$ | 0.273 |
| $LC_{20}$ vs $LC_{40}$ | **0.011** |
| $LC_{30}$ vs $LC_{40}$ | 0.699 |
