# Supplementary material for: Hormetic and transgenerational effects in spotted-wing Drosophila (Diptera: Drosophilidae) in response to three commonly-used insecticides
Source: PLoS One. 2022 Jul 21;17(7):e0271417. doi: 10.1371/journal.pone.0271417 (PMC9302851; doi:10.1371/journal.pone.0271417)
Supplement: S6 Table — Tukey’s post-hoc test P-values for the significant parental treatment*sex interaction on F1 mortality for the pyrethrin treatment. Bolded values indicate statistically significant P-values (P-value ≤ 0.05). (PDF) [file pone.0271417.s007.pdf]

SI Table 6. Treatment*sex interaction post-hoc results for F1 mortality. Tukey's post-hoc test P-values for the significant parental treatment*sex interaction on F1 mortality for the pyrethrin treatment. Bolded values indicate statistically significant P-values (P-value ≤ 0.05).

	treatment by exposure	
contrasts	males	females
LC_0 vs LC_{10}	0.407	1.00
LC_0 vs LC_{15}	1.00	1.00
LC_0 vs LC_{20}	1.00	1.00
LC_0 vs LC_{25}	1.00	1.00
LC_0 vs LC_{30}	1.00	1.00
LC_{10} vs LC_{15}	0.643	0.878
LC_{10} vs LC_{20}	0.515	1.00
LC_{10} vs LC_{25}	1.00	1.00
LC_{10} vs LC_{30}	1.00	1.00
LC_{15} vs LC_{20}	1.00	1.00
LC_{15} vs LC_{25}	1.00	1.00
LC_{15} vs LC_{30}	1.00	1.00
LC_{20} vs LC_{25}	1.00	1.00
LC_{20} vs LC_{30}	1.00	1.00
LC_{25} vs LC_{30}	1.00	1.00
	exposure by treatment	
treatment	males vs females	
LC_0	0.285	
LC_{10}	0.080	
LC_{15}	**0.031**	
LC_{20}	**0.032**	
LC_{25}	0.360	
LC_{30}	0.709	
